# Natural Protein-Restricted Diets and Their Impact on Linear Growth in Patients with Propionic and Methylmalonic Acidemia: A Systematic Review

**DOI:** 10.3390/jpm16010004

**Published:** 2025-12-22

**Authors:** Jessica Ramirez, María Jesús Leal-Witt, Juan Francisco Cabello, Verónica Cornejo

**Affiliations:** Laboratorio de Genética y Enfermedades Metabólicas del Instituto de Nutrición y Tecnología de los Alimentos (INTA), Universidad de Chile, Santiago 7830490, Chile; jessica.ramirez@inta.chile.cl (J.R.); jfcabello@inta.uchile.cl (J.F.C.); vcornejo@inta.uchile.cl (V.C.)

**Keywords:** propionic acidemia, methylmalonic acidemia, protein intake, medical food, failure to thrive

## Abstract

**Background/Objectives:** Propionic acidemia (PA) and methylmalonic acidemia (MMA) affect methionine, threonine, valine (Val), and isoleucine (Ile) (MTVI) metabolism, leading to the production of highly neurotoxic organic acids. Treatment involves a diet restricted in natural proteins and supplemented with a protein substitute (PS) with traces of MTVI. The aim was to analyze natural protein and PS intake in relation to linear growth impairment in individuals with PA and MMA. **Methods:** We followed the PRISMA protocol. We considered articles published between 1970 and 2025. We determined the eligibility criteria for selecting articles and evaluated the quality. **Results**: Thirteen studies were selected: two case reports, eight longitudinal, three cohorts, and one cross-sectional. Articles demonstrated that natural protein intake decreases with age, consistent with previous reports, underscoring the need for PS supplementation to meet protein requirements. Subjects with PA and non-responsive MMA had greater restriction of natural proteins, and the majority required PS; a higher PS intake was negatively correlated with a higher height-for-age (H/A) z-score. When analyzing the ratio of protein to energy (P:E), a negative correlation was found between the intake of natural proteins and energy, and a positive correlation with H/A z-score (*p*-value < 0.05). Supplementation with PS increased leucine levels, causing an imbalance with MTVI amino acids. This imbalance led to the paradoxical need to supplement L-Val and L-Ile, both propiogenic amino acids. As a result, a decrease in the H/A z-score was observed, particularly in PA and non-responsive MMA. Responsive MMA tolerated more natural proteins, received a lower intake of PS, and had a better H/A z-score. **Conclusions:** Restriction of natural proteins and PS is associated with a lower H/A z-score, primarily in subjects with PA and non-responsive MMA.

## 1. Introduction

Organic acidemias are caused by deficiencies of enzymes or cofactors involved in the catabolism of propiogenic amino acids such as methionine (Met), threonine (Thr), valine (Val), and isoleucine (Ile) (MTVI). Propionic Acidemia (PA) is caused by a deficiency of the propionyl-CoA carboxylase (PCC) enzyme, leading to propionic acid accumulation. Methylmalonic acidemia (MMA) is caused by a defect in the mitochondrial enzyme methylmalonyl-CoA mutase (MUT). When the enzymatic defect is complete, the condition is classified as non-responsive MMA (MUT^0^). In contrast, responsive MMA refers to cases in which the defect is partial, with some residual enzymatic activity (MUT^-^), or to cases in which the defect lies in the uptake, synthesis, or transport of the vitamin B12 cofactor (CblA, CblB, CblC). In these cases, vitamin B12 is supplemented, restoring the activity of the mutase enzyme and allowing greater tolerance of natural proteins [1,2]. All forms of MMA cause the accumulation of methylmalonic acid [1,3,4]. It is essential to mention that propionyl-CoA, a key precursor of propionic acid, is derived not only from the catabolism of amino acids (MTVI) but also from the metabolism of odd-chain fatty acids and from propionate produced by intestinal bacterial activity. The implications of these sources are significant, as they require chronic management strategies to ensure metabolic stability [1,4].

Pharmacological management in the chronic phase of PA and MMA aims to minimize the production of toxic organic metabolites, maintain metabolic stability, and support anabolism [1,4]. A key component of this approach is systematic L-carnitine supplementation, which is routinely prescribed in organic acidemias and is widely used in patients with PA (98% of centers) and non-responsive MMA (93% of centers) due to its effectiveness in releasing trapped CoA and preventing secondary carnitine deficiency. Ammonia scavenger drugs are also part of chronic management and are used by 37% of centers for PA and 16% for non-responsive MMA to address hyperammonemia [5,6]

An essential part of the treatment for PA and MMA is nutritional management, which consists of providing a protein-restricted diet that limits natural proteins, particularly those of animal origin, to reduce the intake of propiogenic amino acids [4,7]. Protein restriction does not cover the requirements established by the Recommended Dietary Allowances (RDA) [8]; thus, a particular protein substitute (PS) for these pathologies has been developed [4,9]. The PS is an amino acid mixture that does not contain, or contains only trace amounts of MTVI, has been enriched with leucine (Leu), and has been supplemented with vitamins and minerals, allowing it to meet the RDA for age and sex [8,9,10]. The Leu is added to compete for transfer across the LAT-1 transporter into the blood–brain barrier (BBB) since it has a greater affinity and decreases the entry of MTVI into the brain [10,11]. The Leu is one of the most abundant amino acids in foods. For example, egg protein has an optimal amino acid profile for promoting anabolism, and one gram of egg protein provides approximately 100 mg of Leu. In contrast, PS used in organic acidemias provides about 158 mg of Leu per gram of protein. An imbalance in this PS’s amino acid profile can adversely affect anabolism [12].

It is important to note that Leu’s effects on hypothalamic and brainstem processes related to satiety may contribute to appetite loss in patients with PA and MMA. When exposed to excessive Leu, malnourished individuals may experience reduced growth hormone levels due to an underlying amino acid imbalance [13,14,15,16,17].

Daly and Pinto et al. surveyed within the European Society of Inborn Errors of Metabolism to quantify natural protein intake and the use of PS in subjects with PA and MMA from birth to 16 years of age. They reported that 77% of the centers met the safe protein intake recommendation established by WHO/FAO/UNU (1.0 to 2.2 g/kg/day) [8] and that 81% of the centers used a PS. They also observed that approximately 50% of the total protein requirement was derived from these PS [5,6].

A study in healthy children showed that an intake of 7104 mg of Leu per day, in the absence of Ile and Val (Leu:Ile:Val = 1:0:0), resulted in low protein synthesis. However, when Ile and Val were reintroduced (Leu:Ile:Val = 1:0.26:0.28 and 1:0.35:0.40), optimal protein synthesis was restored [18]. Other studies conducted in individuals with MMA and natural protein restriction reported a decrease in plasma Leu levels, along with reductions in the Leu:Val and Leu:Ile ratios. These changes are thought to result from competition among branched-chain amino acids, both for affinity to the LAT-1 transporter and for tissue utilization [19,20].

Considering the scientific evidence supporting the essential role of dietary treatment in maintaining metabolic stability in PA and MMA, we conducted a systematic review to assess whether restricting natural protein intake and supplementing PS impacts linear growth in individuals with these conditions.

## 2. Materials and Methods

The systematic review was conducted in accordance with the Preferred Reporting Items for Systematic Reviews and Meta-analysis (PRISMA) guidelines [21] (Supplementary Material File S1) and was registered in PROSPERO under the ID 1240154. Our research question, based on the PRISMA protocol, was defined as follows (PICOS): Population (Subjects with Propionic and Methylmalonic acidemias in growth period); intervention (protein intake); comparator (Child growth standard); outcome (z-score height per age); and study design (case reports, observational studies, clinical trials).

**Figure 1:**
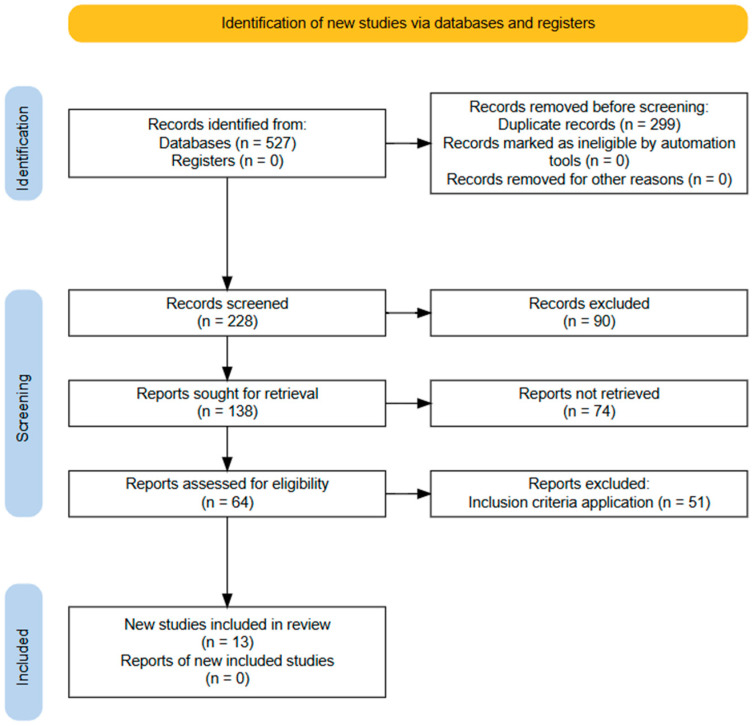
Flow diagram of the information search and collection process in the systematic review.

We conducted research in PubMed, Scielo, Science Direct, and EBSCO databases, considering studies published between 1970 and 2025. Our strategy for searching through the keyword combinations is found in the supplementary material (Supplementary Material File S2), and the selection process and application of exclusion criteria are summarized in Figure 1. All articles were independently reviewed by two reviewers, and a third reviewer was consulted to resolve disagreements or to determine inclusion in cases of uncertainty. The exclusion criteria were studies that included subjects with: pathologies associated with growth; renal failure in subjects with methylmalonic acidemia, other organic acidemias, or liver transplant; pharmacological treatment that could interfere with growth; or subjects who received parental nutritional therapy.

To evaluate linear growth, we used the z-score height-for-age (H/A), a WHO reference that represents the distance between the subject’s height and the median for their age.

The quality of each selected article, except case reports, was assessed through the National Heart, Lung, and Blood Institute Quality Assessment Tool for Observational Cohort and Cross-Sectional Studies (https://www.nhlbi.nih.gov/health-topics/study-quality-assessment-tools) (accessed on 2 February 2024). This tool evaluates 13 items and classifies the article as poor, fair, or good (Supplementary Material File S3) [22].

## 3. Results

Table 1 describes the relevant variables of each selected article. Those that did not accomplish the criteria were excluded (number of articles = 587) (Supplementary Material File S2).

We identified 587 articles, and 13 studies were selected: 2 case reports [23,24], 8 longitudinal studies [25,26,29,30,31,32,33,35], and 3 cohort studies [27,28,34] (Figure 1).

Among the studies reviewed, 9 included subjects diagnosed with PA [27,28,29,30,31,32,33,35]. Similarly, 8 studies examined subjects with non-responsive MMA [23,25,26,27,30,31,34,35], 3 of which specifically included MMA CblA and CblB [25,26,27], and 1 of which included subjects with MMA CblC [28]. This detailed breakdown of the subjects’ conditions provides a clear context for the research findings.

Of the selected articles, 13 provided insights into natural protein intake [23,24,25,26,27,28,29,30,31,32,33,34,35]. Nine studies described the utilization of the PS for supplementation [23,25,27,28,30,31,32,33,34,35]. Four articles expressed the energy intake [25,29,32,35] and calculated the protein:energy ratio (P:E) [29,30,32,35]. Additionally, five articles highlighted the amino acid imbalance [25,27,30,33].

Eight reviewed works considered the H/A z-score as an indicator of growth when investigating ponderal growth [25,27,28,29,30,32,33,34,35].

Satoh et al. [23], in 1981, published a case report about subjects with MMA who received a natural protein intake of 0.6 to 1.2 g/kg/d, which is below the requirement established by the FAO/WHO/UNU for children under two years of age (reference value: 1.6 g/kg/d) [9] and had insufficient weight and height gain. In 1982, Queen et al. [24] reported a 34-month-old case with PA who received a protein intake of 1.0 g/kg/d and was below the 25th percentile for height.

Hauser et al. [25] reported that children (2–9 years old) and adolescents (10–18 years old) with non-responsive MMA (MUT^0^) received restricted natural protein and required substantial supplementation from PS. Despite this, both age groups showed suboptimal growth, as demonstrated by lower H/A z-scores, particularly in girls. Responsive MMA (CblA/CblB) presented slightly higher natural protein intakes and less dependence on PS. Overall energy intake across MMA participants was modest, and several cases required additional Val and Ile supplementation due to low plasma levels [25].

Fujisawa et al. (2013) [26], included 119 cases with MMA (non-responsive and responsive) and PA, and evaluated the natural protein intake in the acute phase (period of metabolic decompensation) and in the chronic phase (period without decompensation), observing that 88% (22/25) of the non-responsive MMA had undergone natural protein restriction in the acute phase and 93% (14/15) in the chronic phase; they reported failure to thrive in 69% of cases. However, in the acute phase, only 39% of responsive MMA required a restriction in natural proteins, 25% in the chronic phase, and only 30% of the total failed to thrive. Concerning PA, 47% had natural protein restrictions in the acute phase and 50% in the chronic phase. Failure to thrive was associated with mortality in patients with MMA [26].

Manoli et al. (2016) [27] found different results; subjects between 2 and 9 years old received a natural protein intake of 1.06 ± 0.29 g/kg/d, and 0.94 ± 0.45 g/kg/d between 10 and 18 years old. They were also supplemented with PS at 0.98 ± 0.68 g/kg/d and 0.72 ± 0.55 g/kg/d, respectively. These subjects had a H/A z-score of −2.07 ± 1.71 SD. Also, they evaluated natural protein intake in 9 subjects with CblA and reported a protein intake of 1.26 ± 0.56 g/kg/d and, among the 6 cases with CblB, an intake of 0.56 ± 0.15 g/kg/d. They found that cases with non-responsive MMA had an H/A z-score of −2.07 ± 1.71 SD, whereas responsive MMA had a z-score of −1.0 ± 1.0 SD. Furthermore, 66% of boys and 25% of girls in this group also had heights below the 10th percentile. They determined that protein intake from the PS showed a non-significant negative trend between Leu/Val and Leu/Ile intake and plasma valine concentration (r = −0.569) and between dietary intake of Leu/Val and height (r = −0.341). This association improved when serum creatinine and insulin-like growth factor-1 (IGF-1) were added to the model, yielding R^2^ values of 0.296 and 0.478, respectively [27].

After that, Manoli et al. (2016) [28] evaluated growth in 28 subjects with MMA, considering CblC cofactor deficiency, of which 14 had congenital microcephaly and seizures, 21% (number of subjects = 6) received less than 85% of the RDA for proteins, and had a H/A z-score of −2.16 ± 1.04 SD. These results are much lower than those of the MMA subjects who received the PS, whose H/A z-score was −1.72 ± 1.01 SD. The authors noted a positive correlation between height and natural protein intake (r = 0.575) [28].

Evans et al. [29] conducted a study on 14 subjects with PA and MMA, of which 50% had an H/A z-score of −1 SD, and the other 50% between −1 and +1 SD. Natural protein intake at 3, 6, and 11 years was 1.5, 1.2, and 0.95 g/kg/d, respectively, making intake comparable with requirements according to the FAO/WHO/UNU. The study also calculated basal metabolic rate using Schofield’s predictive equation, accounting for age, sex, height, and weight. The natural protein-to-energy (P:E) ratio was expressed as grams of protein per 100 calories (kcal)/day (d), within the optimal range of 1.5–2.9 g/100 kcal/d [29].

Molema et al. (2019) [30] analyzed a longitudinal study with 263 subjects who had PA and MMA, whose H/A z-score was classified as standard at birth (−0.52 SD), and who started diet therapy for the diagnosed pathology. An average height of −2 SD was detected at the first medical check-up. Non-responsive MMA were the most compromised (−1.7 SD), despite having 105% protein adequacy according to the RDA and a P:E ratio of 1.23 (a suitable range is 0.37–3.33). They also evaluated plasma levels of MTVI amino acids. They observed that subjects who received only natural protein had lower Val levels (*p* < 0.001). When they received additional PS, they maintained significantly low Val and Ile levels (*p* < 0.001) [30].

Mobarak et al. [31] retrospectively analyzed 20 subjects with PA and MMA. In that case, they used a height percentile and observed that 65% of subjects were below the 10th percentile. They found that lower natural protein was associated with a lower height percentile. Also, they observed a positive correlation between albumin level and natural protein (r = 0.8) and a negative correlation with PS (r = 0.5) [31].

Saleeman et al. [32] evaluated 4 cases during the first five years of life; the authors reported a H/A z-score of −0.72 SD, with a 95% confidence interval of −1.36 to −0.2 SD. The same subjects evaluated between 5 and 10 years had an H/A resulting in a mean of −1.03 SD; after 10 years, the H/A z-score was −1.4 SD. It is important to note that the height of the parents and siblings of the study subjects was within normal ranges. The note indicates that one case received growth hormone treatment at age 9, which improved height. They analyzed protein and energy intake at three years of age, determining a P:E ratio of 2.75 g/100 kcal. When only natural protein intake was considered, this ratio decreased by 0.9 g/100 kcal, well below the optimal reference range (1.5–2.9 g/100 kcal) [29]. The authors concluded that low natural protein intake and a high protein intake from the PS (0.5–1.5 g/kg/day) could be negative factors for linear growth [32].

Mobarak et al. in 2021 [33] evaluated four subjects with PA and observed a total protein intake mean of 1.81 ± 0.51 g/kg/d, of which 0.79 ± 0.15 g/kg/d was natural protein and 1.02 ± 0.49 d/kg/d was PS protein. For subjects whose Val plasma concentration was below the reference range, supplementation with L-Val (180 mg/d) was indicated. Furthermore, they reported that the average intake of Leu was above the RDA (362 ± 165%) and observed a H/A z-score of −1.08 ± 0.96 SD [33].

Molema et al. (2021) [34] evaluated 76 subjects with PA and MMA. They found that 78% maintained a diet restricted in natural protein throughout follow-up and that 37% had intakes above the RDA recommendations, for which PS supplementation had been indicated in 84%. In all forms of MMA and late PA diagnosis, a negative association was found between z-score and restriction of natural proteins, except for early PA diagnosis. By associating the P:E ratio with the H/A z-score, the authors observed a more significant positive association in non-responding MMA [34].

Busiah et al. [35] in 2024 distinguished four periods: early infancy, prepubertal, pubertal, and final height. For each period, they measured each subject’s height. The average of total protein intake in early infancy was 1.1 ± 0.4 g/kg/d with H/A z-score of −0.1 SD, in the prepuberal period was 1.1 ± 0.4 g/kg/d with H/A z-score of −0.3 SD, in the puberal period was 0.8 ± 0.3 g/kg/d with H/A z-score of −1.0 SD, and the final height was H/A z-score of −0.9 SD. In early childhood patients, overall height (SD) correlated positively with the total protein/energy ratio (r = 0.215, *p* = 0.004), but not with IGF1, underscoring the importance of nutrition for growth [35].

## 4. Discussion

The first reports on treatment application were in non-responsive MMA and later in PA [23,24]. Due to nutritional restriction, formulas were manufactured with a low content of MTVI that managed to control acute metabolic decompensation. However, secondarily, a deterioration in linear growth (i.e., an H/A z-score under −1 SD) was observed. This situation is exacerbated with each acute decompensation, as the restriction of natural proteins must be increased. After all, the accumulation of organic acids derived from MTVI triggers a metabolic catastrophe characterized by hyperammonemia [36,37].

It was observed that subjects with PA and non-responsive MMA had a lower H/A z-score than those with responsive MMA, although all these groups evaluated had growth retardation observed with a low H/A, and that increased with age [25,27,29,30,32,33,35].

### 4.1. Protein and Energy Intake Concerning Ponderal Growth

After our analysis, selected studies showed that natural protein intake (g/kg/day) decreases with age in individuals with organic acidemias. This decline does not solely reflect the expected age-related reduction in protein requirements, as outlined in RDA/WHO/FAO guidelines. Instead, it appears to result from a combination of disease-specific metabolic considerations and clinical practice trends in which the stringency of natural protein restriction relative to body weight is maintained or even intensified as patients grow to prevent metabolic decompensation. As a result, in many cases, actual protein intake falls below age-appropriate recommendations, particularly during adolescence and adulthood, potentially contributing to suboptimal growth outcomes [27,29,35]. A European study conducted with 53 centers specialized in the follow-up of subjects with these pathologies observed that 44% and 74% had an intake of natural protein below the recommended safe level in non-responsive MMA and PAs, respectively [5,6,8]. Notably, most PA and non-responsive MMA subjects evaluated had low intake of natural proteins or were within the WHO/FAO/UNU (2007) recommended limit [4,8,9], unlike studies of responsive MMA, which showed greater tolerance to natural proteins, especially those of animal origin.

Natural protein recommendations for a safe minimum intake to maintain growth and development are established quantitatively based on total nitrogen requirements and qualitatively on the amount of essential amino acids and the protein’s digestibility [4,9,38]. The published evidence indicates that natural protein restriction reverses the clinical and biochemical symptoms in the pathologies discussed here. Various case and cohort analyses have been conducted to prevent metabolic decompensation and to avoid deterioration in linear growth caused by low intake of natural proteins [26,30,31]. Thus, it was proposed that diet therapy should be complemented with the PS formulated for these pathologies, based on amino acids, restricted in MTVI, and enriched with Leu [1,4,9]. Since the introduction of PS into long-term diet therapy, new evidence has emerged linking lower intake of natural proteins to PS supplementation and its effect on linear growth, as reflected in the H/A z-score [35,39,40]. The balance between natural protein and protein from PS is essential to decrease adverse effects on linear growth, as reflected by the H/A z-score.

From the studies included in this systematic review, we found that the PS is mainly used among non-responsive MMA and, in most cases, accounts for more than 60% of the total planned proteins [33,34]. Some authors noted that adding PS resulted in 100% of the recommended proteins across age groups being exceeded, and that the contribution of natural proteins remained low or at the limit of the established requirements [5,6,25,27,28,29,32,33]. When determining whether the contribution of natural proteins and that from the PS affected the H/A z-score, six studies observed z-scores below −1 SD, with lower z-scores among non-responsive MMA [29,30,31,32,33,35]. One author found a relationship between higher PS intake and a lower H/A z-score [33].

It should be noted that responsive MMA had a better H/A z-score than the non-responsive MMA [25,27]. That could be because this presentation tolerates more natural protein, as the cofactor’s defects depend on vitamin B12. When supplemented with megadoses of this vitamin, the activity of the mutase enzyme is restored to normal, achieving metabolic stability; hyperammonemia disappears, and the amount of methylmalonic acid is substantially lowered [1,40].

Energy intake is another highly relevant nutritional variable. Protein requirements depend on an adequate energy supply that allows for optimal tissue synthesis and compensates for nitrogen losses, thereby supporting normal growth and metabolic balance. When the diet is deficient in energy, the body uses proteins as an energy source [38,41]. An optimal protein-to-total-energy (P:E) ratio for adequate protein synthesis is 1.5 to 2.9 g/100 kcal, depending on age, body weight, sex, and physical activity [29,42,43]. Different validated equations are used in children, accounting for growth variables, basal energy expenditure, and physical activity [44]. In the studies reviewed, they used the Schofield, WHO/FAO/UNU, and Fleisch equations for subjects under 18 years of age, and the Harris-Benedict and Mifflin-St Jeor equations for subjects older than 18 years [45,46].

Among the studies reviewed, four included an analysis of the P:E ratio, and higher or optimal P:E ratios correlated with better H/A z-scores or optimal growth [29,30,32,35]. Thus, we posit that it is vital to determine the total energy contribution based on the amount of natural protein to prevent the optimal P:E ratio. It is essential to maintain a harmonious protein synthetic process and promote linear growth in diet therapy for these hereditary metabolic pathologies.

Evidence from other inherited amino acid metabolic disorders, such as phenylketonuria (PKU), also supports the notion that dietary management can influence growth trajectories. Evans et al. in 2019 reported that children with PKU frequently present with lower weight-age and H/A z-scores by [4,5] years despite apparently adequate energy and protein intakes, highlighting that dietary composition, not only total intake, may contribute to suboptimal growth [47]. Their study showed that reliance on protein substitutes during the weaning period can reduce natural protein (Phe) intake to very low levels, a factor that may affect growth patterns even when total protein intake meets recommendations. These observations from PKU studies parallel those in PA and MMA, where reduced natural protein intake and a high prescription of protein substitutes may also contribute to impaired linear growth, reinforcing the need to assess both protein quantity and amino acid profile when evaluating growth outcomes in inherited metabolic disorders.

### 4.2. Amino Acids Imbalance

The PS contains traces of MTVI and has been supplemented with Leu, 4–5 times the content of infant formula. Thus, bioavailability and digestibility differ from those of natural protein, which could adversely affect growth due to an imbalance in essential amino acids during this process or reduced availability for protein synthesis [10,38,48]. This generated the hypothesis that PS consumption could alter the relationship between the amino acids Leu/Val or Leu/Ile, which are essential for anabolic processes, and affect linear growth in subjects with PA and MMA [27,32,33].

A recent study in healthy subjects demonstrated that maintaining a balanced ratio of Leu:Val:Ile favors protein synthesis [18]. Three of the articles reviewed showed that subjects presented plasma with low levels of Val, Ile, and Met, leading to the paradoxical need for supplementation with L-Val and L-Ile, both propiogenic amino acids [27,29,32]. When evaluating the cause of this decrease and quantifying the intake of these amino acids, they observed that the intake of PS was the cause of the imbalance between Leu/Met, Leu/Val, and Leu/Ile, obtaining a Leu intake of 362% according to the established recommendations, and the intake of Val and Ile was 91% below the recommendations. Some studies examine whether the imbalance in these amino acids affects growth, focusing on recording their intake and measuring plasma levels. Then, they observed a negative correlation between levels of Val and Ile and linear growth in subjects with MMA, with a higher effect among non-responsive MMA. It was also observed that greater PS intake was negatively correlated with growth, with an H/A z-score of −2 SD [27,30,33]. However, among subjects with responsive MMA who tolerate greater intake of natural proteins, a better result was observed in the H/A z-score [27,28]. Thus, Leu may compete with MTVI amino acids to cross the BBB, considering that the PS has more Leu than international organizations recommend (39 mg/kg/day) [9,10,18]. One study reported a negative correlation between Met and Leu across the BBB (*p* < 0.001) [28]. This finding implies that excessive supplementation of Leu would compete with MTVI amino acids and directly affect the imbalance between them.

Based on our systematic review, which included 13 articles, we can conclude that restricting natural protein intake and increasing PS intake is associated with amino acid imbalance (Met, Ile, and Val). This alteration negatively correlates with the H/A z-score among subjects with PA and non-responsive MMA. Responsive MMA, because they tolerate a more significant number of natural proteins and require a lower or almost null contribution of the PS, achieve a better H/A z-score than those with the non-responsive MMA forms. These studies, along with one excluded research [49] that did not report natural protein intake, support the theory that subjects with organic acidemias have a smaller H/A than children without pathology, which is associated with a higher risk of short stature. It would be interesting to include other propiogenics substrates and their relationship with linear growth in different analyses.

The main limitations of this systematic review are the limited number of scientific publications that assess natural protein intake (particularly from animal sources, considered high-protein), PS, and linear growth in individuals with PA and MMA. The included studies were predominantly longitudinal or cohort follow-ups with small sample sizes, which inherently limit the strength of the inferences. In addition, there was substantial heterogeneity across studies in terms of study design, age ranges, clinical severity, dietary practices, and outcome measures, which limits comparability and reduces the possibility of drawing generalized conclusions. Ethical considerations make double-blind or controlled interventional studies unfeasible in life-threatening metabolic disorders such as PA and MMA, further constraining the available evidence. The cohort studies provided moderate-quality evidence, while the cross-sectional study offered fair-quality evidence. Finally, it was not possible to perform a quality assessment of the case reports due to the lack of a validated evaluation tool for this study type.

One of our exclusion criteria was to exclude studies that considered patients with PA and MMA who had undergone liver transplantation, as liver transplantation fundamentally alters protein tolerance and metabolic control. However, it is interesting to note that in four of the excluded articles [50,51,52,53], improvements in weight and height z-scores post-transplant were observed, mainly due to diet relaxation and increased natural protein intake. Gathering more information to delve deeper into this topic would be interesting.

## 5. Conclusions

Subjects with non-responsive MMA and PA generally showed lower natural protein intake, greater reliance on PS, and lower H/A z-scores. However, these observations are based on a limited number of studies and should therefore be interpreted with caution.

Across the available evidence, natural protein intake (g/kg/day) tended to decrease with age. At the same time, PS consumption was often high enough to result in total protein intakes exceeding RDA recommendations.

Only a few studies reported plasma concentrations of Met, Ile, and Val; therefore, conclusions regarding potential alterations in these amino acids must also be considered preliminary. Although some reports suggest that amino acid imbalances may contribute to the paradoxical need for L-Val and L-Ile supplementation, stronger and more consistent data are required to support this interpretation.

Overall, while the current evidence raises important considerations regarding dietary practices in non-responsive MMA and PA, more robust, homogeneous studies are needed before firm conclusions or clinical recommendations can be established. Nevertheless, these findings support the need for individualized dietary prescriptions that prioritize natural protein intake whenever metabolically feasible.

## Figures and Tables

**Table 1:** Description of articles selected for the systematic review.

Author and Year	Title	Design and Quality	Study Objective	Results	Conclusion
Satoh T, et al., 1981 [23]	Dietary therapy in two patients with vitamin-B_12_ unresponsive methylmalonic acidemia	Case report (*n* = 2) (N/D)	To establish dietary management of vitamin B_12_ among subjects with non-responsive MMA under treatment. Evaluate response to different protein intake.	Case 1 -Total Protein contribution with PS: 0.6, 0.9, and 1.2 g/kg/d, negative changes were observed when the PS was removed, and a better response was obtained to treatment with 1.0 g/kg/d Natural Protein and 1.0 g/kg/d of PS. -PS provided 70–80 kcal/kg/d. Case 2: -Only total protein from Natural Protein without PS, best response with a combination of 1.2 g/kg/d Natural Protein and 1 g/kg/d SP -PS provided 125 kcal/kg/d.	The contribution of PS improved growth, as well as biochemistry, and also presented a significant weight gain. The authors suggest the use of the Natural Protein mixture with PS.
Queen PM, et al. 1982 [24]	The effects of spacing protein intake on nitrogen balance and plasma amino acids in a child with propionic acidemia	Case report (*n* = 1) (N/D)	To evaluate the effect of protein distribution on nitrogen balance and plasma aminogram in a 34-month-old child with propionic acidemia.	H/A percentile: -H/A percentile 10–25 (90.4 cm) -Relationship between NEAA and EAA (r = 4.29 and 4.02) Reference value: (r = 4.0) Total protein intake: 1 g/kg/d	Nitrogen retention was acceptable for linear growth, regardless of protein distribution. Plasma aminogram was adequate for essential amino acids. The authors recommend distributing protein evenly throughout the day.
Hauser NS, et al. 2011 [25]	Variable dietary management of methylmalonic acidemia: metabolic and energetic correlations	Retrospective (*n* = 29) Good	To document different nutritional approaches used to treat patients with MMA, measure estimated energy requirement, and analyze the dependence of estimated energy requirement on body composition, biochemical, and nutritional variables.	H/A z-score: -Non-responsive MMA < 20 y: −0.04 ± 1.2 SD. Females: −1.49 ± −1.4 SD, Males: −0.76 ± 0.9 SD. -Responsive MMA: 0.24 ± 0.9 SD. CblA = 0.78 (males) and −0.84 SD (females) CblB = −1.48 ± 2.7 SD. Natural Protein intake: -Non-responsive MMA Mut^0^ 2–9 y (*n* = 10): 0.84 ± 0.17 g/kg/d 10–18 y (*n* = 07): 0.82 ± 0.46 g/kg/d -Responsive MMA ClbA (*n* = 5): 1.0 ± 0.63 g/kg/d -Responsive MMA CblB (*n* = 2): 0.67 g/kg/d PS intake: -Non-responsive MMA Mut^0^ 2–9 y: 0.72 ± 0.63 g/kg/d 10–18 y: 0.64 ± 0.60 g/kg/d -Responsive MMA ClbA: 0.31 ± 0.52 g/kg/d Energy intake: -MMA (entire group) (38.7 ± 10.7 kcal/kg/d). Low average according to WHO. -MMA (entire group) 2–9 y = 43.1 ± 5.3 kcal/kg/d 10–18 y = 41.2 ± 14 kcal/kg/d -Energy consumption was lower than predicted by the Harris-Benedict and Schofield equations *** -Responsive MMA ClbA: 37.0 ± 9.0 kcal/kg/d -Responsive MMA ClbB: 36.9 ± 19.0 kcal/kg/d Total protein intake: -Average intake: 0.38 ± 2.94 g/kg/d (33% and 98% of natural protein based on RDA). -Non-responsive MMA (*n* = 21): 1.3304 ± 0.72 g/kg/d -Responsive MMA CblA (*n* = 5): 1.32 ± 0.72 g/kg/d -Responsive MMA CblB (*n* = 1): 0.67 g/kg/d R^2^ for protein intake, age, creatinine clearance, and height = 0.66.	Lower energy intake among subjects with MMA. Cases with PS and Natural Protein exceed RDA. Females with responsive MMA had a shorter height than men (H/A z-score < −1 SD). 5 cases had Val and Ile levels below recommended levels and were supplemented.
Fujisawa D, et al. 2013 [26]	Clinical features and organic acidemias in Japan	Retrospective (*n* = 119) Good	To investigate the clinical presentation and evaluate therapies used to improve long-term outcomes in MMA and PA subjects in Japan.	-PA: (*n* = 19) 47% natural protein restriction in the acute phase and 50% in the chronic phase. -Non-responsive MMA (*n* = 25) had a restriction of 88% in Natural Protein in the acute phase and 93% in the chronic phase; 69% presented failure to thrive. -Responsive MMA (*n* = 15) had 38.5% restriction of Natural Protein in the acute phase and 25% in the chronic phase; 30% presented failure to thrive. -70% of the non-responsive MMA had failure to thrive, and 30% in responsive MMA and PA. -Failure to thrive was considered a mortality factor in all forms of MMA (*p* < 0.001) -95% of subjects evaluated received L-carnitine supplementation.	Non-responsive MMA have a lower survival rate. Growth impairment is directly affected by Natural Protein restriction.
Manoli, I. et al. 2016 [27]	A critical reappraisal of dietary practices in methylmalonic acidemia raises concerns about the safety of medical foods. Part 1: isolated methylmalonic acidemias	Cohort (*n* = 61) Good	To evaluate the effects of unbalanced BCAA intake on metabolic and growth parameters in a cohort of patients with MMA.	H/A z-score: -Non-responsive MMA: Mut^0^ (*n* = 28) = −2.07 ± 1.71. (Height < 10th percentile: 66% of men and 25% of women) Mut^-^, Responsive MMA CblA and CblB =< −1 SD. Total Protein and Natural Protein intake: -Non-responsive MMA Mut^0^: 0.99 ± 0.32 g/kg/d (102 ± 30% RDA). -MMA: 2–9 y = 2.04 ± 0.81 g/kg/d (1.06 g/kg/d) (105% RDA). 10–18 y = 1.67 ± 0.73 g/kg/d (0.94/kg/d) (99.8% RDA). -Responsive MMA ClbA: 1.58 ± 0.89 g/kg/d (1.26 ± 0.56 g/kg/d Natural Protein) -Responsive MMA ClbB: 1.04 ± 0.29 g/kg/d (0.56 ± 0.15 g/kg/d Natural Protein) PS intake: -Non-responsive MMA (all): 0.78 ± 0.68 g/kg/d 2–9 y = 0.98 ± 0.68 g/kg/d. 10–18 y = 0.72 ± 0.55 g/kg/d. -Responsive MMA CblA = 0.31 ± 0.49 g/kg/d. CblB = 0.47 ± 0.40 g/kg/d. Amino acid intake: -Leu/Val or Leu/Ile was significantly higher in patients with PS. -Leu intake: Non-responsive MMA (all) 2–9 y = 222.0 ± 24.9 mg/d 10–18 y = 173.33 ± 55.6 mg/d	The PS provides between 4 and 5 times more Leu than recommended by WHO/FAO/UNU 2007 [8], and was negatively correlated with growth. Significant negative correlation between protein from PS intake and plasma concentration of Val r = −0.569 and Ile r = −0.469. Significant negative correlation between Leu/Val intake and height in patients with non-responsive H/A z-score and Mut^0^: r = −0.341 (*n* = 23) and R^2^ = 0.123. After adding serum creatinine factors to the model, the correlation improved to R^2^ = 0.296 and IGF-1 to R^2^ = 0.478.
Manoli I, et al. 2016 [28]	A critical reappraisal of dietary practices in methylmalonic acidemia raises concerns about the safety of medical foods. Part 2: cobalamin C deficiency	Cohort (*n* = 28) Good	To examine the effects of an unbalanced intake of branched-chain amino acids on growth in patients with CblC-responsive MMA.	H/A z-score: -Mean = −1.04 ± 1.33 SD 14 subjects with congenital microcephaly and seizures = −2.16 ± 1.04 SD. Natural Protein Intake: 21% of subjects receive less than 85% of RDA. PS intake: 32% of subjects who received PS had H/A z-score = −1.72 ± 1.01 SD.	A greater relationship was observed between the intake of Leu with Met or with Val, when they were with PS, and a negative correlation was detected with H/A z-score (r = −0.673; *p* = 0.033) Positive correlation between Natural Protein intake and H/A z-score: r = 0.575 Negative correlation of Met entry through the BBB compared to Leu ***.
Evans M, et al. 2017 [29]	The relationship between dietary intake, growth, and body composition in inborn errors of intermediary protein metabolism	Longitudinal retrospective (*n* = 14) Good	To examine the relationship between protein and energy (P:E) intake and growth in patients with inborn errors of metabolism, and determine a safe P:E ratio for optimal growth.	H/A z-score: 50% of subjects with PA/MMA = H/A z-score less than −1 SD, the other 50% between −1 and +1 SD. Natural Protein intake: -Decreases with age (1.5 y = 1.2 g/kg/d) (3, 6 and 11 y = 0.95 g/kg/d) -Negative correlation between Natural Protein intake and energy (*n* = 6) r = −0.522, *p* = 0.0288. -A positive correlation was observed between Total Protein intake and weight (*n* = 10; r = 0.56; *p* = 0.08), but not so with height. -Negative correlation between Natural Protein intake and the lowest fat% (*n* = 11; r = −0.74 **). -A correlation was obtained between the P:E ratio between 1.5 and 2.9 gr prot/100 kcal/day with optimal growth, BMI, and % fat	Despite adequate protein and energy intake, growth outcomes in patients with inborn errors of intermediary protein metabolism are not always ideal. They also show that a P:E ratio range of >1.5–<2.9 g protein/100 kcal/day correlates with optimal growth, BMI, and fat mass % in those with inborn errors of intermediary protein metabolism.
Molema F, et al. 2019 [30]	Decreased plasma L-arginine levels in organic acidurias (MMA and PA) and decreased plasma branched-chain amino acid levels in urea cycle disorders as a potential cause of growth retardation: Options for treatment.	Longitudinal (*n* = 263) Good	To study the correlation between the level of L-arginine, BCAA, and height among patients with PA/MMA and in urea cycle disorders.	H/A z-score at birth: -PA/MMA = −0.52 SD (range −5.53 to 3.48). First appointment post-diagnosis: 33% had a H/A z-score less than −2.0 SD. -In non-responsive MMA Mut^0^ = −1.7 ± 1.56 SD Natural protein intake: -Greater protein restriction in symptomatic individuals (Z = −2.38 *) and in those receiving PS (Z = −2.087 *). At the first visit, the average caloric prescription was 105% of the RDA, and in relation to the Natural Protein:E ratio, it was 1.23 (range: 0.37–3.33), and Total Protein:E was 1.83 (range: 0.63–4.56). Natural Protein intake with using PS: -Average natural protein intake prescription of 110% (range: 18–278%) of RDA with the PS. Plasma amino acids: -Decrease in the plasma level of Ile and Val in the first control. -Val level was < in subjects with Natural Protein restriction *** -Val and Ile levels were lower in subjects receiving the PS ***.	PA/MMA: positive association between plasma levels of L-Arg and L-Val, Natural Protein:E ratio, and H/A z-score. In the multilevel analysis, a positive correlation was observed between H/A z-score and the ratio of Natural Protein: E ***, and a negative correlation with the amount of PS ** and the age at visit ***. Authors suggest optimizing levels of these amino acids to improve the Natural Protein:E ratio and promote adequate growth.
Mobarak A, et al. 2020 [31]	Clinical course and nutritional management of Propionic and Methylmalonic Acidemias	Retrospective (*n* = 20) Good	Provide information on clinical response and long-term complications by identifying possible factors correlated with complications.	10 PA and 10 MMA were analyzed. Average height percentile of 10.85 ± 6.71; 65% were below the 10th percentile. Total protein intake: -Total group (*n* = 20) 2.09 ± 0.24 g/kg/d of which 0.73 ± 0.1 g/kg/d was natural protein and 1.37 ± 0.24 g/kg/d was PS. -PA (*n* = 10) 2.08 ± 0.28 g/kg/d of which 0.71 ± 0.1 g/kg/d was natural protein and 1.38 ± 0.26 g/kg/d was PS. -MMA (*n* = 10) 2.12 ± 0.2 g/kg/d of which 0.75 ± 0.1 g/kg/d was natural protein and 1.36 ± 0.23 g/kg/d was PS. 2 cases had an intake under the RDA and had a height in the 3rd percentile. -Albumin level correlated positively with Natural Protein (r = 0.8 ***) and negatively with the PS (r = −0.48 *). -The levels of both albumin and prealbumin were positively correlated with Natural Protein.	A greater consumption of PS was observed, and a lower intake of Natural Protein was associated with a lower height percentile. It is suggested that individuals may have a better outcome with a higher intake of Natural Protein. PS should only be used in cases where patients do not meet 100–120% of their RDA.
Saleemani H, et al. 2021 [32]	Dietary management and growth outcomes in children with propionic acidemia: A natural history study	Longitudinal retrospective (*n* = 4) Good	To describe the protein and caloric intake and the long-term impact on the growth of four patients with PA.	H/A z-score: 0–5 y: average −0.72 SD (−1.36 to −0.2 SD). 5–10 y: average −1.03 SD (−1.78 to −0.23 SD) 10–18 y: average −1.4 SD. -One case was treated with growth hormone at 9 years old. Parent height: -Father > 1.70 m and mother > 1.6 m. -Expected height and actual height achieved at age 18, 3/4 below expected; 1 patient died before age 18. Sibling height: 2 brothers of 2 subjects evaluated: H/A z-score from 0 to 1 SD. Energy intake (Total Protein:E ratio): -Total Protein:E = 2.75 g/100 kcal. -Natural Protein: E = 0.9 g/100 kcal.	Although caloric and Total Protein intake covered the RDA, the H/A z-score was lower than expected. Natural Protein intake was lower, with a Natural Protein:E ratio lower than recommended. Optimal dietary management is suggested for PA, balancing the use of Natural Protein and PS, using the Natural Protein:E ratio.
Mobarak A., et al. 2021 [33]	Long-term follow-up of the dietary intake in propionic acidemia	Longitudinal retrospective (*n* = 4) Good	To provide a detailed view of the dietary intake, plasma amino acid profiles, and long-term growth parameters of 4 subjects with PA.	H/A z-score: -H/A z-score was the most affected, with an average of −1.08 ± 0.96 SD (range −6.1 to 1.81) Total protein intake (natural + PS): -Total Protein intake = 1.81 ± 0.51 g/kg/d (182 ± 50% above RDA) -Natural Protein = 0.79 ± 0.15 g/kg/d (80 ± 13% adequacy with RDA) -PS = 1.02 ± 0.49 g/kg/d (103 ± 49% adequacy according to RDA). Amino acid intake: -Average supplementation of 180 mg/d Val (17 ± 9%) due to low plasma levels. -Average Met intake under RDA recommendations and remained low in two evaluation points (1–4 y: 204.34 ± 71.27 mg/d; and 4–7 y: 355.76 ± 94.18 mg/d; d). -Average Leu intake = 362 ± 165% above the RDA. -High Leu/Val and Leu/Ile ratio. Plasma amino acid level: -Decreased levels of Val and Ile by 91% and 42%, respectively (Val: 67.41 ± 33.05 μmol/L; Ile: 49.79 ± 25.67 μmol/L)	Despite an increased intake of Total Protein and amino acids, an imbalance was observed in the ratios of BCAA, so it is suggested to monitor them. Use PS only in patients who do not meet RDAs for Natural Protein and monitor plasma amino acid levels.
Molema F, et al. 2021 [34]	High protein prescription in methylmalonic and propionic acidemia patients and its negative association with long-term outcome	Retrospective Cohort (*n* = 76) Fair	To evaluate the association of longitudinal dietary treatment, in relation to Natural Protein, PS, and Total Protein, with episodes of metabolic decompensation, long-term mitochondrial complications, cognitive development, and height.	H/A z-score: -Cohort (*n* = 76): most participants showed impaired growth. -Non-responsive MMA (*n* = 24): negative association with age and a positive association with a higher P:E ratio. Total protein intake: -Cohort: 150 ± 52% of the RDA. The RDA was exceeded in 84% of assessments. -Non-responsive MMA: 149 ± 48% of the RDA. The RDA was exceeded in 80%. -Responsive MMA: 141% of the RDA. The RDA was exceeded in 79%. -PA, early diagnosis: 154 ± 49% of the RDA. The RDA was exceeded in 91%. -PA, late diagnosis: 142% of the RDA. The RDA was exceeded in 81%. -A higher Total Protein:E ratio was associated with a greater H/A z-score in subjects with non-responsive MMA. Natural protein intake: 78% had a diet restricted in Natural Protein throughout the follow-up. -Cohort: 37% exceeded the RDA for natural protein. -Non-responsive MMA: the RDA was exceeded in 34%. -Responsive MMA: the RDA was exceeded in 53%. -PA, early diagnosis: the RDA was exceeded in 26%. -PA, late diagnosis: the RDA was exceeded in 44%. PS intake: 84% received the PS. -Higher PS intake was negatively associated with H/A z-score in all except subjects with early diagnosed PA. -MMA group: High prescription was associated with more mitochondrial complications.	The prescription of Natural Protein was above the RDA; however, subjects still received the PS. High protein intake was negatively associated with metabolic decompensation, cognitive development, and height. It is suggested to adapt the protein prescription according to the RDA, especially in the most severe forms, and provide PS when there is a deficiency.
Busiah K., et al. 2024 [35]	Pubertal origin of growth retardation in inborn errors of protein metabolism: A longitudinal cohort study	Longitudinal retrospective (*n* = 89) Good	Inherited aminoacid metabolism disorders (IAAMDs) require a lifelong protein-restricted diet. We aimed to investigate: 1/whether IAAMDs were associated with growth, pubertal, bone mineral apparent density, or body composition impairments; And associations linking height, PS, plasma amino acids, and IGF-1 concentrations.	H/A z-score: Early infancy (0–4 y): average −0.1 SD (1.3 SD). 7.7% < −2 SD. Prepubertal (4–8 y girls and 4–9 boys): average −0.3 SD (1.7 SD). 14.9% < −2 SD. Pubertal: average −1.0 SD. 31% < −2 SD. Total protein intake (natural + PS): -Total Protein intake = Early infancy 1.1 ± 0.4 g/kg/d (100 ± 38.8% above RDA); Prepubertal 1.1 ± 0.4 g/kg/d (106.4 ± 37.6% above RDA); Pubertal 0.8 ± 0.3 g/kg/d (94.8 ± 33.5% above RDA) -Natural Protein = Early infancy 0.9 ± 0.4 g/kg/d (78.9 ± 40.6% adequacy with RDA); Prepubertal 0.9 ± 0.3 g/kg/d (85.7 ± 32.6% adequacy with RDA); Pubertal 0.7 ± 0.3 g/kg/d (78.4 ± 34.3% adequacy with RDA). -PS = Early infancy 0.7 ± 0.2 g/kg/d; Prepubertal 0.5 ± 0.2 g/kg/d; Pubertal 0.4 ± 0.2 g/kg/d. Energy intake (Total Protein:E ratio): -P:E = Early infancy 1.3 ± 0.4 g/100 kcal; Prepubertal 1.5 ± 0.5 g/100 kcal; Pubertal 1.9 ± 0.7 g/100 kcal.	In early patients, overall height (SD) correlated positively with total protein/energy ratio, but not with IGF1.

Abbreviations. BBB: blood–brain barrier; BCAA: branched-chain amino acid; BMR: basal metabolic rate; cm: centimeter; E: energy; EAA: essential amino acids; g: gram; g/kg/d: gram/kilogram weight/day; H/A: height/age; Ile: isoleucine; L: length; L/A: length/age; Leu: leucine; Met: methionine; mg/d: milligram/d; MMA: methylmalonic acidemia; *n* = number of subjects studied; NEAA: non-essential amino acids; non-responsive MMA: non-responsive methylmalonic acidemia (Mut^0^); PA: propionic acidemia; PS: protein substitute; r: ratio; RDA: daily requirement; responsive MMA: Responsive methylmalonic acidemia (Mut^-^); cofactor deficiency (CblA, CblB or CblC); SD: standard deviation; μmol/L: microgram/liter; Val: valine; WHO: World Health Organization. %: percentage; * *p* < 0.05, ** *p* < 0.01, *** *p* < 0.001.

## Data Availability

The original contributions presented in this study are included in the article/Supplementary Material. Further inquiries can be directed to the corresponding author.

## Supplementary Materials

The following supporting information can be downloaded at: https://www.mdpi.com/article/10.3390/jpm16010004/s1, File S1: PRISMA 2020 checklist; File S2: Strategies to Articles selection; File S3: National Heart, Lung, and Blood Institute Quality Assessment Tool for Observational Cohort and Cross-Sectional Studies.

## Abbreviations

The following abbreviations are used in this manuscript:

**Abbreviation**	**Definition**
Met Thr	Methionine Threonine
Val	Valine
Ile	Isoleucine
Leu	Leucine
MTVI	Methionine, Threonine, Valine, Isoleucine
PA	propionic acidemia
MMA	methylmalonic acidemia
PCC	Propionyl-CoA carboxylase
MUT^0^	Non-responsive MMA
MUT^-^	Responsive MMA
MMA CblA, CblB, CblC	Cofactor deficiency of the mutase enzyme
RDA	Recommended Dietary Allowances
PS	Protein substitute protein
BBB	Blood–brain barrier
LAT-1	Transporter of large, branched, and aromatic neutral amino acids
PRISMA	Preferred Reporting Items for Systematic Reviews and Meta-analysis
PICOS	The acronym is used to help formulate a well-defined research question. P: population; I: Intervention/exposure; C: comparison; O: outcome; S: Study design
SD	Standard deviation
H/A	Height per age
P:E	Protein energy and Energy ratio
IGF-1	Insulin-like growth factor-1
